# Expression Profiles of Mitochondrial Genes in the Frontal Cortex and the Caudate Nucleus of Developing Humans and Mice Selectively Bred for High and Low Fear

**DOI:** 10.1371/journal.pone.0049183

**Published:** 2012-11-13

**Authors:** Kwang H. Choi, Thien Le, Jennifer McGuire, Jennifer Coyner, Brandon W. Higgs, Suad Diglisic, Luke R. Johnson, David M. Benedek, Robert J. Ursano

**Affiliations:** 1 Department of Psychiatry, Center for the Study of Traumatic Stress, Uniformed Services University of the Health Sciences, Bethesda, Maryland, United States of America; 2 Elashoff Consulting, Redwood City, California, United States of America; 3 Stanley Medical Research Institute, Rockville, Maryland, United States of America; Wayne State University, United States of America

## Abstract

A growing body of evidence suggests that mitochondrial function may be important in brain development and psychiatric disorders. However, detailed expression profiles of those genes in human brain development and fear-related behavior remain unclear. Using microarray data available from the public domain and the Gene Ontology analysis, we identified the genes and the functional categories associated with chronological age in the prefrontal cortex (PFC) and the caudate nucleus (CN) of psychiatrically normal humans ranging in age from birth to 50 years. Among those, we found that a substantial number of genes in the PFC (115) and the CN (117) are associated with the GO term: mitochondrion (FDR qv <0.05). A greater number of the genes in the PFC (91%) than the genes in the CN (62%) showed a linear increase in expression during postnatal development. Using quantitative PCR, we validated the developmental expression pattern of four genes including monoamine oxidase B (*MAOB*), NADH dehydrogenase flavoprotein (*NDUFV1*), mitochondrial uncoupling protein 5 (*SLC25A14*) and tubulin beta-3 chain (*TUBB3*). In mice, overall developmental expression pattern of *MAOB, SLC25A14* and *TUBB3* in the PFC were comparable to the pattern observed in humans (p<0.05). However, mice selectively bred for high fear did not exhibit normal developmental changes of *MAOB* and *TUBB3*. These findings suggest that the genes associated with mitochondrial function in the PFC play a significant role in brain development and fear-related behavior.

## Introduction

A substantial number of genes in the brain undergo developmental changes in psychiatrically normal subjects [1], [2], [3]. Many genes implicated in psychiatric disorders exhibit dynamic expression changes during the first decade of life [1]. Thus, it is likely that disruption of normal expression pattern of the susceptibility genes during development may contribute to the development of psychiatric symptoms in adulthood. Animal studies have shown that adolescence is a sensitive period for the development of stress and anxiety responses in adulthood [4], [5]. For example, repeated exposure of rats to a stressor across an adolescent period increase fearfulness in a novel environment in adulthood and resulted in lower levels of dopamine receptor subtype-2 levels in the prefrontal cortex (PFC) [6]. One of the potential mechanisms may include different hypothalamus-pituitary-adrenal (HPA) axis responses to stressors in young and adult animals [7], [8]. A slow maturation of the PFC toward adulthood may contribute to different stress responses in animals [9]. These studies implicate a functional relationship between brain development, stress and altered fear behavior.

The PFC is considered as one of the most functionally advanced regions of the human cortex [10], mediating working memory, response inhibition and management of autonomic control [11], [12]. The PFC has been implicated in the pathophysiology of psychiatric disorders including schizophrenia, mood and anxiety disorders [13], [14], [15], [16]. Thus, disruption of the PFC function during normal brain development may contribute to the increased likelihood of developing psychiatric disorders in adulthood [10], [17], [18]. In contrast, the caudate nucleus (CN), a part of the basal ganglia, has been implicated in motor control, stimulus response and habit learning [19], [20]. The CN receives synaptic inputs from the dorsolateral PFC [21], [22] and may also be involved in cognitive dysfunction of schizophrenia [23], [24]. However, the CN has received much less attention despite the fact that the CN had more genes differentially expressed than the PFC in individuals with schizophrenia [25].

Mitochondria generate energy as adenosine triphosphate (ATP) and are involved in the apoptosis-signaling pathway [26]. Hundreds of nuclear genes and a few dozen mitochondrial genes coordinate complex mitochondrial function such as intracellular ATP and calcium buffering, oxidative phosphorylation, synaptic activity and apoptosis. Mitochondrial dysfunction has been implicated in a variety of pathological conditions including developmental disorders in primates [27], [28], [29], [30]. Furthermore, somatic deletions of mitochondrial DNA (mtDNA) may be associated with development and aging [31], [32]. A spectrum of somatic mutations in mtDNA may be due to oxidative damage during normal aging. For instance, the breakdown of dopamine by mitochondrial monoamine oxidase B (MAOB) produces H_2_O_2_ which can lead to oxygen radical formation [33]. This may increase the spectrum of somatic mutations produced by oxidative damage. Thus, brain regions that are involved in dopamine metabolism such as the prefrontal cortex (PFC) and the caudate nucleus (CN) may be particularly vulnerable to oxidative damage. Previous studies reported developmental patterns of gene expression in the PFC of human brain tissue [2], [34], [35]. However, expression profiles of mitochondrial genes were not described in those studies. A study reported that approximately 20 genes associated with the mitochondrial membrane are enriched in developing human brains based on the template matching procedure and the gene set enrichment analysis [36]. A comprehensive expression profile of mitochondrial genes in the PFC and the CN has not been reported.

Mitochondrial genes are implicated in psychiatric disorders including schizophrenia [37], bipolar disorder [38], major depression [39], anxiety disorder [40], and posttraumatic stress disorder [41]. For example, a significant decrease in mitochondrial ATP production and mitochondrial enzyme activity was found in individuals with major depression [42]. Also, genes associated with mitochondrial function and immune responses were differentially expressed in the individuals with bipolar disorder and major depression [43]. Furthermore, genes involved in energy metabolism and mitochondrial function were down-regulated [38], [44] and genes involved in immune response and inflammation were up-regulated in bipolar disorder patients [45], [46]. One of the major modulators of mitochondrial function is *BCL-2* which is embedded in the inner mitochondrial membrane. Transgenic mice over-expressing *Bcl-2* in the brain showed a decrease in anxiety and neophobia [47], whereas *Bcl-2* knockout mice showed a significant increase in anxiety-like behavior [40], suggesting the involvement of *Bcl-2* in anxiety disorders. Together, these studies indicate that the genes associated with mitochondria may play a significant role in mood and anxiety disorders.

Although mitochondrial dysfunction during development may contribute to the development of mood and anxiety disorders [40], [48], [49], [50], molecular mechanisms by which mitochondrial genes influence brain development and fear-related behavior remain unclear. Using microarray data available from the public domain and Gene Ontology analysis, we surveyed genes and functional categories associated with age in the cortical (PFC) and the sub-cortical (CN) areas of psychiatrically normal subjects ranging in age from birth to 50 years. Expression profiles of different genes from this microarray dataset have been published [1], [2], [35], [36], and we are testing a novel hypothesis using mitochondria-associated genes. Using mice selectively bred for high and low fear, we investigated the effects of age and altered fear behavior on mitochondrial gene expression. To our knowledge, this is the first study reporting developmental expression patterns of mitochondrial genes in different brain regions and altered fear responses. Our study demonstrates the utility of integrating the expression data derived from postmortem brain tissue of psychiatrically normal individuals and a mouse model of fear to enhance our understanding of the mitochondrial function in brain development and fear-related disorders.

## Results

### Age-related Genes in the PFC and the CN

Individual variable analyses revealed that brain pH affected expression of a significant number of transcripts: 6.6% of the transcripts in the PFC and 0.24% of the transcripts in the CN. Other demographic variables such as postmortem interval (PMI) (PFC: 1.9% and CN: 0.08%), RNA Integrity Number (RIN) (PFC: 1.1% and CN: 0.3%), race (PFC: 0.4% and CN: 0.1%) and sex (PFC: 0.1% and CN: 0.1%) affected a relatively small number of transcripts. Thus brain pH was adjusted using a multiple regression model. We identified genes showing linear changes across age such as 1,236 genes (716 increase and 520 decrease) in the PFC and 1,745 genes (985 increase and 760 decrease) in the CN based on the significance criteria (r^2^>0.6 and qv <0.05) (Figure S1). Using those age-related genes, we performed Gene Ontology (GO) analyses and found the same GO term: mitochondrion that is enriched in both the PFC (115 genes, fold change: 1.96, FDR <5%) and the CN (117 genes, fold change: 1.4, FDR <5%) as shown in Table 1.

**Table 1:** Enriched biological pathways in the genes showing age-dependent changes in the PFC and the CN of normal individuals.

Brain Region	Category	GO Term	Count	Fold Change	FDR p-value
PFC	GOTERM_CC_ALL	GO:0005739∼mitochondrion	115	1.96	3.98E-10
PFC	GOTERM_CC_ALL	GO:0031966∼mitochondrial membrane	58	2.62	3.99E-09
PFC	GOTERM_BP_ALL	GO:0006119∼oxidative phosphorylation	28	4.32	8.18E-07
PFC	GOTERM_CC_ALL	GO:0005746∼mitochondrial respiratory chain	22	4.87	9.87E-08
PFC	GOTERM_BP_ALL	GO:0007399∼nervous system development	86	1.96	4.66E-06
CN	GOTERM_BP_ALL	GO:0007242∼intracellular signaling cascade	176	1.42	0.002793
CN	GOTERM_BP_ALL	GO:0000074∼regulation of progression through cell cycle	75	1.72	0.003894
CN	GOTERM_BP_ALL	GO:0007399∼nervous system development	101	1.56	0.005043
CN	GOTERM_CC_ALL	GO:0005739∼mitochondrion	117	1.4	0.009768
CN	GOTERM_BP_ALL	GO:0008219∼cell death	103	1.49	0.012914

Functional annotation analyses (Gene Ontology) were performed using 2 sets of genes (genes changing expression in the PFC and genes changing expression in the CN). Count: number of genes included in each category, Fold: fold enrichment, FDR: false discovery rate-adjusted p-values based on the Benjamini-Hochberg method [84].

### Expression of Mitochondrial Genes in the PFC

**Figure 1:**
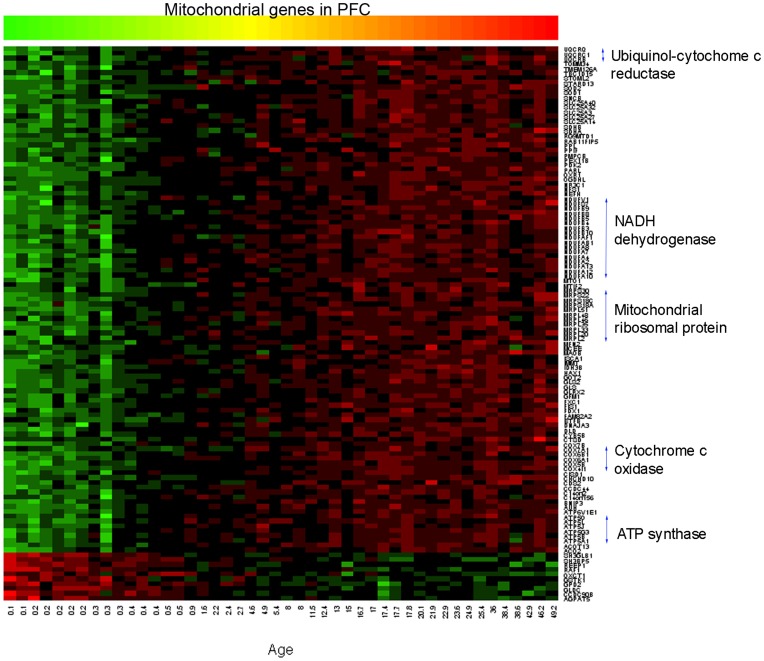
Developmental expression pattern of the genes associated with mitochondrial function in the PFC. A majority of the mitochondrial genes (91%) show increased expression (green to red), while only 9% of the genes show decreased expression (red to green) during postnatal development. Genes that encode different subunits of the same protein are shown on the right side. X-axis: Age (years). Y-axis: Gene symbols. In this pseudo-color heat map, increasing red intensities indicate genes with high expression levels, and increasing green intensities indicate genes with low expression levels across age. Color bar scale: hybridization intensity (log base 2) from 2.41 to 11.72.

A majority of the genes associated with the GO term: mitochondrion in the PFC (105/115 genes, 91%) showed a linear increase in expression during postnatal development (Figure 1). Among those, multiple genes encode different subunits of the same protein that are involved in the oxidative phosphorylation function (Table S1). For example, 17 genes encode sub-complexes of the NADH dehydrogenase (*NDUF*), 6 genes encode the ATP synthase (*ATP5*), 6 genes encode the cytochrome c oxidase (*COX*) and 3 genes encode the ubiquinol-cytochrome c reductase (*UQCR*) as shown on the right side of Figure 1. These suggest that a demand for energy synthesis and metabolism in the PFC gradually increases during postnatal development.

### Expression of Mitochondrial Genes in the CN

**Figure 2:**
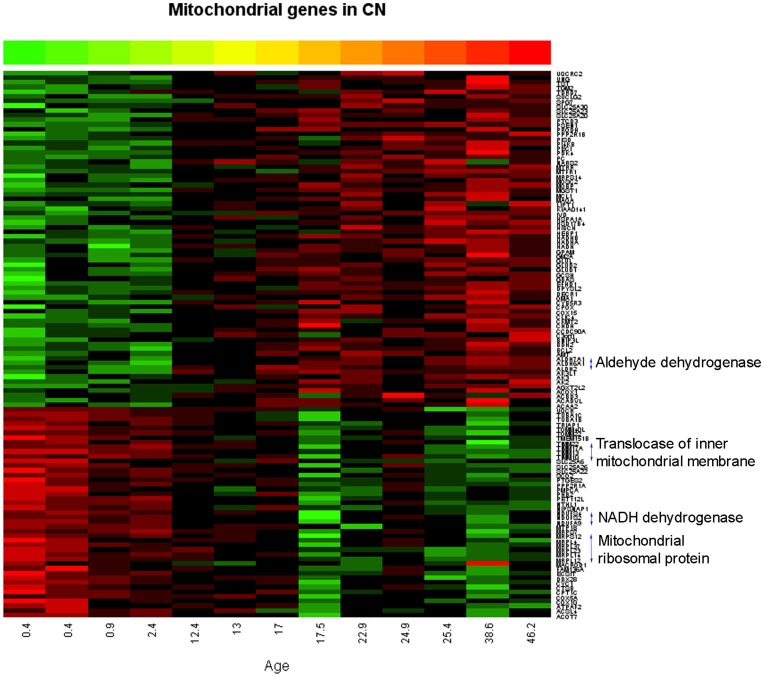
Developmental expression pattern of the genes associated with mitochondrial function in the CN. Approximately 62% of the genes (72/117) show increasing expression (green to red), while 38% of the genes (45/117) show decreasing expression (red to green) during development. X-axis: Age (years). Y-axis: Gene symbols. Genes that encode different subunits of the same protein are shown on the right side. On the contrary to the PFC, very few genes encode different subunits of the same protein. Color bar scale: hybridization intensity (log base 2) from 4.45 to 13.56.

Although an overall number of age-related genes associated with the GO term: mitochondrion was similar between the PFC (115) and the CN (117), individual gene expression patterns were quite different. While a majority of the genes in the PFC (91%) showed a linear increase with age, less number of the genes in the CN (62%) showed the same pattern with age (Figure 2). On the contrary to the age-related genes in the PFC (43), fewer genes (17) in the CN encode different subunits of the same protein as shown on the right side of Figure 2.

### Quantitative PCR

**Figure 3:**
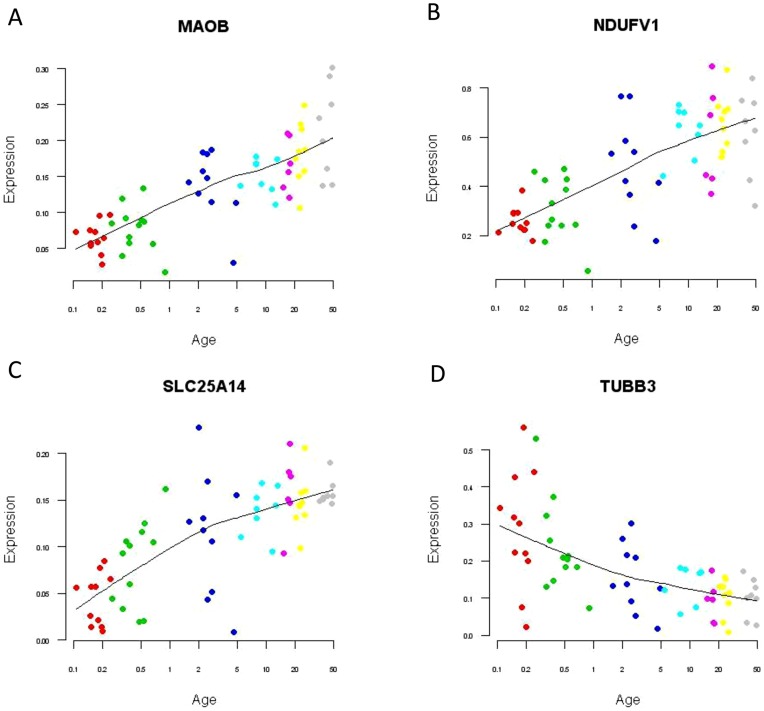
Quantitative PCR validation of mitochondrial genes. A scatter plot with a line of best fit demonstrates that each gene in the PFC shows either increase or decrease in expression across age (qv <0.05). X-axis: Age (log 2 scale). Y-axis: Gene expression (log 2 scale). Subjects were color-coded as red: neonate (0–3 months), green: infant (3–12 months), blue: toddler (1–5 years), light blue: school age (6–13 years), pink: teenage (14–19 years), yellow: young adult (20–30 years), and grey: adult (31–50 years). A: *MAOB* (monoamine oxidase B), B: *NDUFV1* [NADH dehydrogenase (ubiquinone) flavoprotein 1, 51 kDa], C: *SLC25A14* (mitochondrial uncoupling protein 5), D: *TUBB3* (tubulin beta-3 chain).

Using quantitative PCR, we validated the developmental expression patterns of four genes including monoamine oxidase B (*MAOB*), NADH dehydrogenase (ubiquinone) flavoprotein (*NDUFV1*), mitochondrial uncoupling protein 5 (*SLC25A14*) and tubulin beta-3 chain (*TUBB3*) in the PFC. We selected these genes because they are included in the list of 115 genes from the GO term: mitochondrion and have been implicated in psychiatric disorders: monoamine oxidase B [51], [52], NADH dehydrogenase (ubiquinone) flavoprotein [53], [54], mitochondrial uncoupling protein 5 [55], and tubulin [56], [57]. We have confirmed that the microarray and the qPCR data were consistent, with all four genes showing the same directional changes in both experiments. We used the multiple regression analysis including brain pH as a covariate, and calculated the adjusted coefficient (r^2^) and adjusted p-value for each gene. Expression levels of *MAOB* (r^2^ = 0.55, qv = 7.5E-10), *NDUFV1* (r^2^ = 0.63, qv = 1.8E-08), and *SLC25A14* (r^2^ = 0.53, qv = 3.1E-05) gradually increased while the levels of *TUBB3* (r^2^ = 0.51, qv = 4.3E-06) decreased across chronological age (Figure 3).

### Expression of Mitochondrial Genes in Mice with High and Low Fear

**Figure 4:**
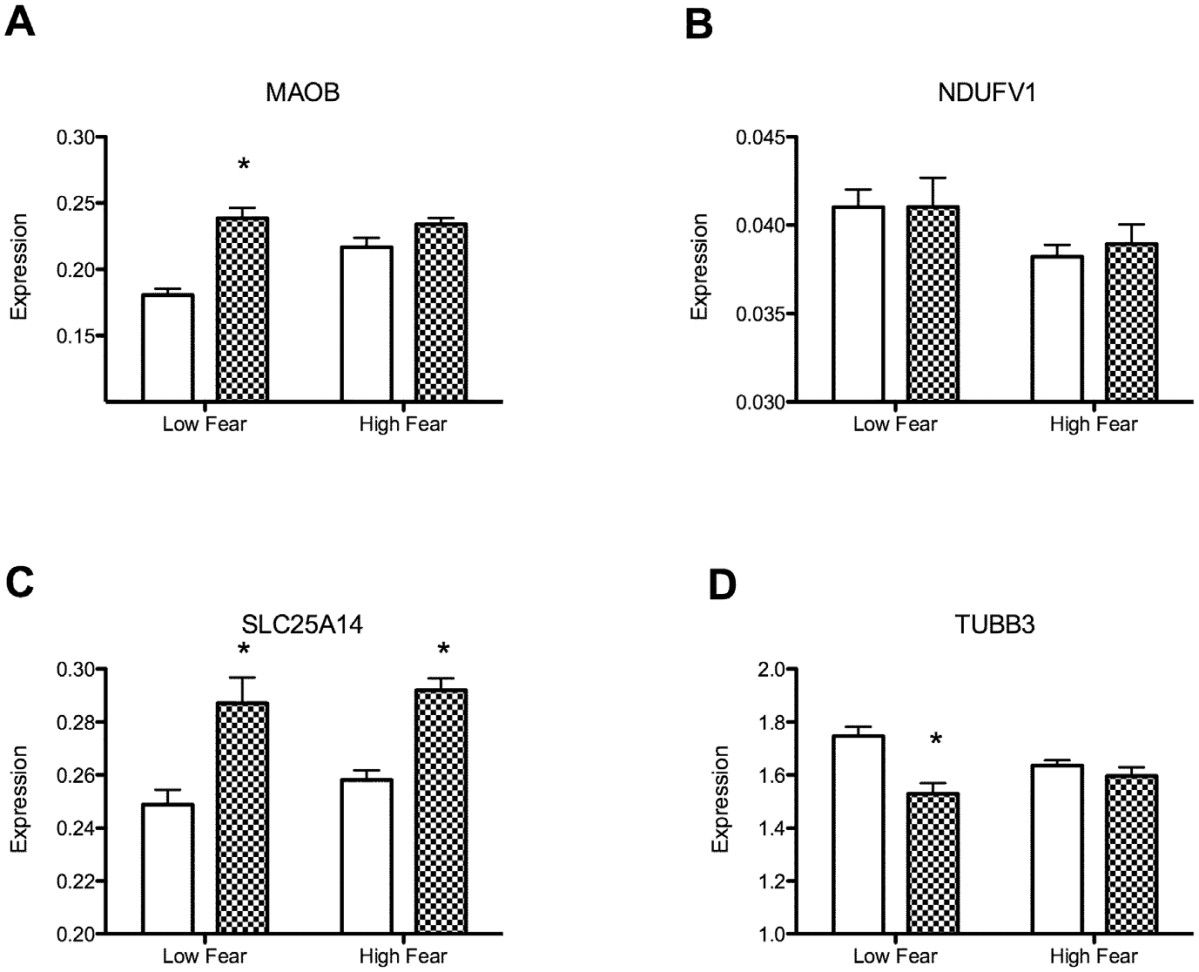
Expression levels of mitochondrial genes in the PFC of juvenile and adult mice selectively bred for high and low fear. The expression levels of mitochondrial genes in the PFC of mice selectively bred for high and low fear were measured in either 1 month (clean bar) or 4 months (hatched bar) of age. A: *MAOB* (monoamine oxidase B), B: *NDUFV1* [NADH dehydrogenase (ubiquinone) flavoprotein 1, 51 kDa], C: *SLC25A14* (mitochondrial uncoupling protein 5), D: *TUBB3* (tubulin beta-3 chain). Data shown as an average and SEM. *Significant between juvenile and adult mice (p<0.05).

We investigated the effects of age and fear behavior in mice selectively bred for high and low fear. We quantified the expression levels of the same genes including *MAOB, NDUFV1, SLC25A14* and *TUBB3* in the PFC of juvenile and adult mice selectively bred for high and low fear (Figure 4). For *MAOB*, there was a significant interaction between age and fear (F [_1_], [_79_] = 8.68, p<0.05). A post-hoc analysis revealed significant effects between juvenile and adult mice selectively bred for low fear (Figure 4A). Expression levels of *NDUFV1* were not different between these groups (p>0.05) as shown in Figure 4B. Expression levels of *SLC25A15* were higher in adult mice as compared to juvenile mice (p<0.05) as shown in Figure 4C. For *TUBB3*, a significant interaction between age and fear was found (F [_1_], [_79_] = 7.88, p<0.05). Among the low fear mice, the levels of *TUBB3* were lower in adult mice as compared to juvenile mice (p<0.05) (Figure 4D). These results indicate that the mice selectively bred for low fear exhibit normal developmental expression pattern of those genes. However, the mice selectively bred for high fear exhibit disrupted expression patterns of *MAOB* and *TUBB3* in the PFC during postnatal development.

## Discussion

A normal mitochondrial function is critical for synaptogenesis and spine formation [58], [59], and for normal apoptosis to occur [60], [61]. Thus, increased expression of the genes associated with mitochondrial function in the PFC during development may reflect ongoing maturation and neuronal plasticity, especially during adolescence [36]. For instance, *MAOB* is present on the outer membrane of the mitochondria and function primarily to maintain the cytosolic concentrations of monoamines. The precise spatial and temporal pattern of the monoamine neurotransmitter systems is known to be important in orchestrating the development of the neural circuitry of the brain [62], [63], [64]. Consequently, the metabolism of the monoamines by *MAOB* in the developing brain is going to be fundamental for brain development and function. Given that *MAOB* expression levels gradually increase in the PFC during normal brain development, a lack of developmental changes in *MAOB* levels observed in high fear mice indicates a dysfunction of *MAOB* in these animals. This is an important finding because *MAOB* has been implicated in mood and anxiety disorders including social phobia, panic disorder and post-traumatic stress disorder (PTSD) [65]. Thus, enhancing *MAOB* activity in the PFC may have beneficial effects on fear-related behavior. Our findings support the notion that monoamines are involved in mood and anxiety disorders.

We found that the genes associated with the GO term: mitochondrion undergo age-related changes in expression in both the PFC and the CN of developing humans. However, only the genes from the PFC showed a consistent increase in expression across age. Also there were more genes in the PFC than in the CN that are involved in oxidative phosphorylation function. A growing body of evidence suggests mitochondrial dysfunction in affective disorders involving multiple brain regions, including the PFC [44], the temporal cortex [66], and the hippocampus [38]. Moreover, base pair substitutions in the coding regions of mtDNA [67], altered mitochondrial oxidative phosphorylation [68] and abnormal expression of nuclear genes encoding mitochondrial proteins [38] have been reported in mood and anxiety disorders. These results strongly implicate mitochondrial dysfunction in the pathophysiology of affective disorders [50]. In line with these findings, the major categories of drugs used to treat depression have been demonstrated to exert effects on mitochondria as well as on monoamines [69], [70], [71]. Also, commonly used mitochondrial-targeted treatments exert effects on mitochondria and are increasingly being shown to demonstrate efficacy in mood disorders [72]. These studies suggest an interaction between the monoamine system and the mitochondrial system in mood and anxiety disorders.

Although the mitochondrial system has been implicated in psychiatric disorders, very little is known about the role of mitochondrial genes on fear learning in rodents. We investigated the expression levels of four mitochondrial genes in the PFC of mice selectively bred for high and low fear. The classical fear conditioning model has been used extensively to study fear in animals [73] and in humans [74]. We have found that three mitochondrial genes (*MAOB, SLC25A14* and *TUBB3*) in the PFC follow age-dependent changes in expression in mice selectively bred for low fear. However, normal developmental changes of *MAOB* and *TUBB3* were disrupted in mice selectively bred for high fear. This is significant because mice selectively bred for high fear resemble individuals who are more susceptible to develop fear-related disorders [75]. Thus, disrupted expression levels of *MAOB* and *TUBB3* in the PFC of mice with high fear may contribute to exaggerated fear responses observed in these animals.

A limitation of this study is that we had a relatively smaller number of postmortem brain samples from the CN (n = 14) as compared to the PFC (n = 48), so the statistical power may be compromised. However, we observed a similar number of genes associated with mitochondrial function in the PFC (115 genes) and the CN (117 genes) using the same criteria of significance (r^2^>0.6 and FDR q-value <0.05). It is possible that other factors such as nutrition, metabolism or common deletions in mtDNA associated with aging may have affected expression of certain mitochondrial genes. It would be important to corroborate the current gene expression findings from developing brains with other types of data such as brain imaging, neuropsychological and cognitive testing to enhance our understanding on human brain development and function. Another limitation is that a limited number of mitochondrial genes were tested in the PFC of mice with high and low fear. Thus it is possible that other mitochondrial genes that were not investigated in this study may also contribute to fear and anxiety behavior. Also, we did not study effects of stress on fear behavior in these animals and a further study is necessary to expand the current findings.

In summary, we identified a substantial number of genes associated with mitochondria that undergo age-dependent changes in the PFC and the CN of psychiatrically normal individuals. A majority of the genes in the PFC (105/115) showed a linear increase in expression across age and 27% of them (28/105) were related to oxidative phosphorylation function. Using mice selectively bred for high and low fear, we found that age-dependent changes in expression of *MAOB* and *TUBB3* in the PFC were disrupted in animals with high fear. Since mitochondrial dysfunction can lead to multiple abnormalities in cell function [76], [77], disruptions in normal developmental changes of the genes during the sensitive period may predispose the individuals to the development of mood and anxiety disorders. Taken together, a better understanding of the genes associated with the mitochondrial function in the PFC may provide an opportunity to identify a novel drug target for the treatment of mood and anxiety disorders.

## Materials and Methods

### Postmortem Brain Tissue and Microarray Experiment

Postmortem brain tissue from the PFC (Brodmann Area 46) and dorsal head of the CN ranging in age from birth to 50 years were obtained from the National Institute of Child Health and Development Brain and Tissue Bank for Developmental Disorders (NICHD Contract NO1-HD8-3283; IRB approval H-20765) (Table S2). Details on sample collection and consent information is available from a previous report [78]. Brain tissue from the CN was not available from all the subjects, and this resulted in much fewer samples included in the microarray experiments (PFC: 48 samples and CN: 14 samples) (Figure S2). The brain collection protocol was reviewed and approved by the Institutional Review Board of the University of Maryland, Baltimore. All subjects were free of neurological and psychiatric symptoms at the time of death as described previously [79]. A microarray experiment (Affymetrix HG-U133 plus 2.0 GeneChip) was performed by Dr. Paabo’s group (Max Planck Institute, Germany) and findings from this dataset were published previously [2], [35], [36].

### Quality Control of Microarrays

Raw data (.cel files) were processed and analyzed using the R statistical language (http://www.r-project.org) and Bioconductor packages [80]. A robust multi-array average (RMA) algorithm was used for normalization of expression values (log base 2) for each transcript [81]. Microarray data quality was assessed using a pair-wise sample correlation coefficient with hierarchical clustering to identify sample outliers. Transcripts were filtered out if 20% or more of the subjects had expression values of less than a 1.1-fold change in either direction from the transcript’s median value and if the percent of subjects with an absent gene call exceeded 33% using the Affymetrix calls. We used this procedure to remove transcripts that are not expressed or changed across the samples before the statistical analysis [82]. After the gene filtering, 21,391 transcripts for the PFC and 22,356 transcripts for the CN were retained.

### Microarray Data Analysis

First, individual demographic factors were analyzed to identify potential confounding factors affecting the expression of a significant number of genes. The number of transcripts significantly regulated by each variable including brain pH, postmortem interval (PMI), RNA integrity number (RIN), race and sex was calculated using a linear regression model (p<0.001). Following the demographic factor analysis, linear gene expression changes across chronological age were analyzed in a series of multiple regression models, one model for each gene, including age (log base 2) and brain pH (as a confounder) as independent variables and gene expression (log base 2) as a dependent variable. To adjust for multiple testing of the genes, the calculated p-values corresponding to the age covariate for each gene were adjusted to give an overall false discovery rate (FDR) of 5% using the q-value package (www.bioconductor.org). The criteria of significance were set at adjusted coefficient r^2^>0.6 and FDR q-value <0.05. The microarray data are available from the Gene Expression Omnibus (GEO) under the accession number GSE11512.

### Gene Ontology Analysis

The NCBI's Database for Annotation, Visualization and Integrated Discovery (DAVID; http://david.abcc.ncifcrf.gov/) was used as a standard source for gene annotation information [83]. A modified Fisher’s Exact test (EASE) was used to measure the gene set enrichment in the annotation terms. A set of genes associated with age in each brain region was used in an annotation term-by-annotation term contingency test to identify the association between each gene set and annotation term. Both nominal and FDR adjusted p-values for each test were calculated, and the significance threshold for the GO term was set at FDR-adjusted p<0.05 [84].

### Mice Selectively Bred for High and Low Fear

Mice were derived from the F_8_ generation of C57BL/6J (B6) X DBA/2J (D2) advanced intercross line (AIL). The foundation AILs were created and tested by Dr. Abraham Palmer and colleagues (University of Chicago, Chicago IL) [85], [86], [87]. The F_8_ AILs were trained and tested for cued and contextual fear [88], and mice that display either enhanced (top 20%) or diminished (bottom 20%) conditioned fear (selected generation 1) were shipped to the Uniformed Services University of the Health Sciences where breeding and selection continued. Thus, these animals represent alternative condition as compared to the animals in the middle that were not selected for breeding. Juvenile (approximately 1 month) and adult (approximately 4 months) mice from the third and the fourth generations that display high and low fear were used in this experiment including juvenile mice with high fear (N = 31) and low fear (N = 19), and adult mice with high fear (N = 21) and low fear (N = 12). Animals were screened for high and low fear using Pavlovian fear conditioning. Mice were placed in Plexiglas rodent conditioning chambers with a metal grid floor (Ugo Basile, Collegeville, PA) and a single house light provided dim lighting within sound attenuation boxes. Mice were given a 3 min baseline to adjust to the context and then presented with two 30 sec tones (conditioned stimuli [CS], 5 kHz, 75 dB) that terminated with a mild electric foot shock (unconditioned stimuli [US], 0.5 sec, 0.8 mA) using the AnyMaze program (Stoelting Co., Wood Dale, IL). The following day, animals were placed in the identical chamber with no tones or shocks presented and freezing in response to the test chamber (contextual fear) was measured for 5 min. For cue-specific fear, the context of the chamber was changed, which included covering the house light with yellow acetate film. Striped and checkered patterns were placed around the Plexiglas and a white plate was placed over the metal grid floor. With the change in context, the mice were tested on freezing in response to the tone (CS). The mice were monitored with infrared cameras, which measured freezing time for each animal during the testing (ANY-maze program, Stoelting Co., Wood Dale, IL). These animals showed clear differences in conditioned fear behavior as reported previously [89]. For example high fear and low fear mice exhibited approximately 55% and 30% freezing behavior respectively during CS presentation. An animal protocol was approved by the IACUC at the Uniformed Serviced University, Bethesda, MD.

### RNA Extraction from Mouse Brain Tissue

Coronal sections of 1.5 mm mouse brain slices were acquired using an acrylic brain block (Braintree Scientific, Braintree, MA) and surgical razor blades on wet-ice (4°C). The medial PFC was punched out using a 14-gauge needle, and immediately frozen in dry ice. Brain tissue was homogenized by ultrasonication and total RNA was extracted using the RNeasy Minikit (Qiagen, Valencia, CA, USA). Complementary DNA was synthesized using a reverse-transcriptase polymerase chain reaction (RT-PCR) using oligo dT primers.

### Quantitative PCR

Total RNA was extracted from the PFC of the same subjects as described in postmortem brain tissue section above, and the cDNA was synthesized with RT-PCR using oligo dT primers. Pre-designed and validated QuantiTect SYBR primers (Qiagen, Valencia, CA, USA) were used for the qPCR: *MAOB* (QT00009870, NM_000898), *NDUFV1* (QT00003080, NM_007103), *SLC25A14* (QT00040544, NM_003951), and *TUBB3* (QT00083713, NM_006086). Three endogenous control genes including *PP1A* (QT01866137, NM_021130), *GUSB* (QT00046046, NM_000181) and *ACTB* (QT00095431, NM_001101) were used. For mouse brain tissue, oligonucleotide primers were designed using the Primer 3 software (http://frodo.wi.mit.edu/primer3/). Primer sequences were *Maob* (forward: cagccagaaccagaatctttg, reverse: gctgacaagatggtggtcaat), *Ndufv1* (forward: cgttgactggatgaacaaggt, reverse: gtgtggccttctatctgcttg), *Slc25a14* (forward: tgaatcagagggcaatagtgg, reverse: atgatgttccagggtccaagt) and *Tubb3* (forward: gaatgacctggtgtccgagta, reverse: cgattcctcgtcatcatcttc). Using a 384-well format with the Prism 7900HT real-time detector (Applied Biosystems, Foster City, CA), 1 µl aliquots of Qiagen QuantiTect SYBR primer, 10 µl qPCR PCR Master mix (Applied Biosystems, Foster City, CA), and 10 µl cDNA were mixed together and pipetted into single wells of the qPCR plate. Reactions were quantified by the cycle threshold (Ct) method using the SDS2.2 software (Applied Biosystems, Foster City, CA). An average quantity value (Qty mean) for each sample from the triplicates of that sample was calculated for each gene. The data for each gene were expressed as Qty mean for the gene of interest/geometric mean of Qty mean for the three endogenous control genes. Multiple regression analyses were performed with chronological age (log base 2) and brain pH as covariates as described previously [79]. For the mice data, a two way ANOVA (age X fear) for interaction and main effects followed by *post-hoc* tests were performed using normalized values of the individual genes.

## Supporting Information

Figure S1 **Distribution of actual age across samples (PFC: n = 46 and CN: n = 13).** There were more samples with age below 10 (25 out of 46 samples) in the PFC as described in the demographic summary table. In order to better describe the expression changes during early development, we used a log2 scale of age in Figure 3.
(DOCX)

Figure S2 **A Venn diagram showing the total number of genes with age-related expression changes between the PFC and the CN (r^2^>0.6 and FDR qv <0.05).** There were 1,236 genes (716 increased and 520 decreased) in the PFC and 1,745 genes (985 increased and 760 decreased) in the CN that undergo age-related changes in expression.
(DOCX)

Table S1 **A summary of demographic information.** PMI: Postmortem interval, RIN: RNA integrity number M: Male, F: Female, AA: African American, C: Caucasian
(DOCX)

Table S2 **Information on the genes associated with the GO term: mitochondrion in the PFC and the CN.** r^2^: adjusted coefficient, r: regression coefficient, q value: FDR-adjusted q-value
(DOCX)
