# Supplementary material for: Expression Profiles of Mitochondrial Genes in the Frontal Cortex and the Caudate Nucleus of Developing Humans and Mice Selectively Bred for High and Low Fear
Source: PLoS One. 2012 Nov 13;7(11):e49183. doi: 10.1371/journal.pone.0049183 (PMC3496717; doi:10.1371/journal.pone.0049183)

Figure S2. Distribution of actual age across samples (PFC: n=46 and CN: n=13). There were more samples with age below 10 (25 out of 46 samples) in the PFC as described in the demographic summary table. In order to better describe the expression changes during early development, we used a log2 scale of age in Figure 3.


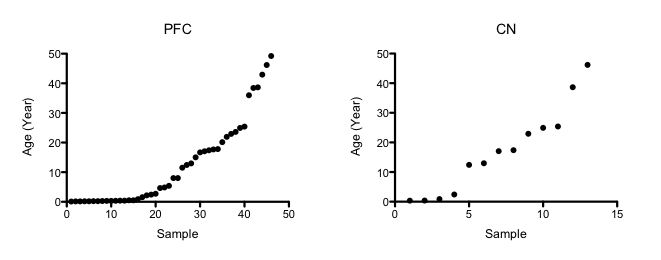

Supplement: Figure S2 — A Venn diagram showing the total number of genes with age-related expression changes between the PFC and the CN (r2>0.6 and FDR qv <0.05). There were 1,236 genes (716 increased and 520 decreased) in the PFC and 1,745 genes (985 increased and 760 decreased) in the CN that undergo age-related changes in expression. (DOCX) [file pone.0049183.s002.docx]
