# Supplementary material for: Expression Profiles of Mitochondrial Genes in the Frontal Cortex and the Caudate Nucleus of Developing Humans and Mice Selectively Bred for High and Low Fear
Source: PLoS One. 2012 Nov 13;7(11):e49183. doi: 10.1371/journal.pone.0049183 (PMC3496717; doi:10.1371/journal.pone.0049183)
Supplement: Table S1 — A summary of demographic information. PMI: Postmortem interval, RIN: RNA integrity number M: Male, F: Female, AA: African American, C: Caucasian (DOCX) [file pone.0049183.s003.docx]

Table S1. Information on the genes associated with the GO term: mitochondrion in the PFC and the CN.

r^2^: adjusted coefficient, r: regression coefficient, q value: FDR-adjusted q-value

PFC

| **Gene ID** | **Gene Symbol** | **r^2^** | **r** | **p value** | **q value** |
| --- | --- | --- | --- | --- | --- |
| 27089 | UQCRQ | 0.610 | 0.781 | 1.6651E-08 | 3.33157E-08 |
| 7384 | UQCRC1 | 0.659 | 0.812 | 2.19681E-09 | 5.36293E-09 |
| 7381 | UQCRB | 0.615 | 0.784 | 1.2503E-09 | 3.22693E-09 |
| 10953 | TOMM34 | 0.605 | 0.778 | 2.56408E-08 | 4.92455E-08 |
| 84233 | TMEM126A | 0.602 | 0.776 | 1.06096E-06 | 1.42556E-06 |
| 64786 | TBC1D15 | 0.702 | 0.838 | 3.90302E-12 | 1.76622E-11 |
| 30968 | STOML2 | 0.601 | 0.775 | 1.44752E-07 | 2.36685E-07 |
| 90627 | STARD13 | 0.661 | 0.813 | 6.08694E-10 | 1.67824E-09 |
| 6648 | SOD2 | 0.745 | 0.863 | 8.11598E-14 | 5.76466E-13 |
| 6647 | SOD1 | 0.817 | 0.904 | 6.26834E-15 | 6.22489E-14 |
| 6620 | SNCB | 0.728 | 0.853 | 1.43602E-12 | 7.2097E-12 |
| 55972 | SLC25A40 | 0.613 | 0.783 | 2.82593E-08 | 5.36462E-08 |
| 81034 | SLC25A32 | 0.656 | 0.810 | 5.5332E-10 | 1.53969E-09 |
| 5250 | SLC25A3 | 0.688 | 0.830 | 9.04652E-11 | 2.99827E-10 |
| 9481 | SLC25A27 | 0.669 | 0.818 | 2.25801E-09 | 5.5024E-09 |
| 9016 | SLC25A14 | 0.665 | 0.815 | 9.54916E-10 | 2.52108E-09 |
| 6390 | SDHB | 0.659 | 0.812 | 1.23462E-08 | 2.55235E-08 |
| 6389 | SDHA | 0.772 | 0.879 | 9.23332E-14 | 6.43979E-13 |
| 54931 | RG9MTD1 | 0.612 | 0.782 | 1.70102E-09 | 4.26218E-09 |
| 26056 | RAB11FIP5 | 0.792 | 0.890 | 8.92654E-15 | 8.4292E-14 |
| 5493 | PPL | 0.753 | 0.868 | 2.55202E-12 | 1.20001E-11 |
| 10105 | PPIF | 0.602 | 0.776 | 1.35041E-09 | 3.44913E-09 |
| 9512 | PMPCB | 0.696 | 0.834 | 3.68345E-11 | 1.32227E-10 |
| 8799 | PEX11B | 0.627 | 0.792 | 3.66826E-08 | 6.79679E-08 |
| 5164 | PDK2 | 0.767 | 0.876 | 3.8744E-13 | 2.22602E-12 |
| 55486 | PARL | 0.762 | 0.873 | 4.5503E-14 | 3.45403E-13 |
| 55074 | OXR1 | 0.794 | 0.891 | 3.98098E-14 | 3.0824E-13 |
| 55753 | OGDHL | 0.922 | 0.960 | 1.26533E-24 | 2.44581E-22 |
| 2908 | NR3C1 | 0.823 | 0.907 | 2.76875E-16 | 4.16796E-15 |
| 9054 | NFS1 | 0.754 | 0.868 | 6.93302E-14 | 5.02317E-13 |
| 4744 | NEFH | 0.829 | 0.910 | 9.55803E-17 | 1.59661E-15 |
| 4723 | NDUFV1 | 0.639 | 0.799 | 9.3341E-08 | 1.59062E-07 |
| 374291 | NDUFS7 | 0.741 | 0.861 | 3.9674E-12 | 1.79386E-11 |
| 4715 | NDUFB9 | 0.779 | 0.883 | 9.02171E-14 | 6.3248E-13 |
| 4714 | NDUFB8 | 0.907 | 0.953 | 3.98013E-23 | 5.12889E-21 |
| 4711 | NDUFB5 | 0.718 | 0.848 | 1.78629E-12 | 8.70187E-12 |
| 4710 | NDUFB4 | 0.876 | 0.936 | 6.35947E-19 | 2.0128E-17 |
| 4709 | NDUFB3 | 0.636 | 0.797 | 9.44545E-09 | 2.00933E-08 |
| 4716 | NDUFB10 | 0.758 | 0.870 | 4.25136E-13 | 2.41949E-12 |
| 51103 | NDUFAF1 | 0.624 | 0.790 | 9.30161E-08 | 1.58609E-07 |
| 4706 | NDUFAB1 | 0.629 | 0.793 | 3.15385E-08 | 5.92275E-08 |
| 4702 | NDUFA8 | 0.788 | 0.888 | 5.37394E-14 | 4.00862E-13 |
| 4701 | NDUFA7 | 0.710 | 0.842 | 6.16774E-12 | 2.66183E-11 |
| 4697 | NDUFA4 | 0.611 | 0.782 | 4.99295E-08 | 9.01666E-08 |
| 4695 | NDUFA2 | 0.660 | 0.812 | 1.28574E-08 | 2.64224E-08 |
| 51079 | NDUFA13 | 0.619 | 0.787 | 1.95429E-08 | 3.85743E-08 |
| 55967 | NDUFA12 | 0.765 | 0.875 | 1.5609E-13 | 1.01294E-12 |
| 4705 | NDUFA10 | 0.603 | 0.777 | 1.2193E-08 | 2.5248E-08 |
| 25821 | MTO1 | 0.671 | 0.819 | 9.97561E-10 | 2.62854E-09 |
| 4528 | MTIF2 | 0.707 | 0.841 | 1.86433E-12 | 9.0576E-12 |
| 10884 | MRPS30 | 0.644 | 0.803 | 3.81084E-10 | 1.10001E-09 |
| 56945 | MRPS22 | 0.810 | 0.900 | 6.53701E-15 | 6.39779E-14 |
| 51023 | MRPS18C | 0.799 | 0.894 | 1.71659E-13 | 1.09429E-12 |
| 55168 | MRPS18A | 0.603 | 0.777 | 2.72942E-09 | 6.51619E-09 |
| 51258 | MRPL51 | 0.836 | 0.915 | 8.085E-17 | 1.39214E-15 |
| 51642 | MRPL48 | 0.718 | 0.847 | 4.09253E-10 | 1.17318E-09 |
| 26589 | MRPL46 | 0.609 | 0.780 | 6.14493E-08 | 1.08721E-07 |
| 51318 | MRPL35 | 0.689 | 0.830 | 1.53923E-10 | 4.87742E-10 |
| 9553 | MRPL33 | 0.809 | 0.900 | 3.34624E-14 | 2.67118E-13 |
| 55052 | MRPL20 | 0.670 | 0.819 | 2.17233E-11 | 8.27385E-11 |
| 51069 | MRPL2 | 0.645 | 0.803 | 2.50351E-10 | 7.56534E-10 |
| 9927 | MFN2 | 0.615 | 0.784 | 1.41715E-09 | 3.61108E-09 |
| 84693 | MCEE | 0.616 | 0.785 | 8.5466E-08 | 1.46845E-07 |
| 4129 | MAOB | 0.749 | 0.865 | 4.31972E-14 | 3.30215E-13 |
| 81689 | ISCA1 | 0.629 | 0.793 | 7.33011E-08 | 1.27933E-07 |
| 10989 | IMMT | 0.619 | 0.787 | 5.97256E-08 | 1.05948E-07 |
| 3420 | IDH3B | 0.763 | 0.873 | 1.93793E-12 | 9.36474E-12 |
| 10456 | HAX1 | 0.797 | 0.893 | 2.50514E-14 | 2.08912E-13 |
| 2806 | GOT2 | 0.847 | 0.920 | 9.0946E-17 | 1.53819E-15 |
| 27165 | GLS2 | 0.736 | 0.858 | 6.40488E-12 | 2.75992E-11 |
| 2744 | GLS | 0.777 | 0.882 | 1.66124E-13 | 1.06908E-12 |
| 51022 | GLRX2 | 0.830 | 0.911 | 2.40719E-16 | 3.70121E-15 |
| 85476 | GFM1 | 0.686 | 0.828 | 1.83487E-11 | 7.08831E-11 |
| 26515 | FXC1 | 0.700 | 0.836 | 8.41173E-12 | 3.53464E-11 |
| 51024 | FIS1 | 0.809 | 0.899 | 5.92376E-15 | 5.91525E-14 |
| 2230 | FDX1 | 0.623 | 0.789 | 1.02116E-08 | 2.15635E-08 |
| 55177 | FAM82A2 | 0.840 | 0.916 | 4.31608E-17 | 7.91849E-16 |
| 2109 | ETFB | 0.685 | 0.827 | 1.00938E-11 | 4.17024E-11 |
| 9093 | DNAJA3 | 0.728 | 0.853 | 5.80437E-11 | 2.01117E-10 |
| 1738 | DLD | 0.635 | 0.797 | 4.95794E-09 | 1.11806E-08 |
| 80777 | CYB5B | 0.632 | 0.795 | 1.01861E-09 | 2.68008E-09 |
| 1509 | CTSD | 0.663 | 0.814 | 3.23677E-11 | 1.18126E-10 |
| 1349 | COX7B | 0.605 | 0.778 | 1.41954E-08 | 2.87855E-08 |
| 1346 | COX7A1 | 0.694 | 0.833 | 1.66686E-11 | 6.50426E-11 |
| 1340 | COX6B1 | 0.785 | 0.886 | 1.26626E-14 | 1.15375E-13 |
| 1337 | COX6A1 | 0.654 | 0.809 | 3.79843E-09 | 8.78545E-09 |
| 1329 | COX5B | 0.815 | 0.903 | 8.20445E-16 | 1.03748E-14 |
| 1327 | COX4I1 | 0.879 | 0.937 | 8.52131E-20 | 3.57513E-18 |
| 55847 | CISD1 | 0.638 | 0.798 | 1.01637E-07 | 1.72002E-07 |
| 400916 | CHCHD10 | 0.877 | 0.937 | 1.15116E-20 | 6.23033E-19 |
| 8760 | CDS2 | 0.601 | 0.775 | 5.12914E-09 | 1.15091E-08 |
| 51204 | CCDC44 | 0.708 | 0.842 | 4.23345E-12 | 1.89046E-11 |
| 9556 | C14orf2 | 0.734 | 0.857 | 1.65901E-11 | 6.4783E-11 |
| 81892 | C14orf156 | 0.617 | 0.785 | 4.22401E-06 | 4.96984E-06 |
| 664 | BNIP3 | 0.638 | 0.798 | 3.95115E-10 | 1.13747E-09 |
| 549 | AUH | 0.730 | 0.854 | 1.67061E-12 | 8.20482E-12 |
| 529 | ATP6V1E1 | 0.868 | 0.931 | 2.16237E-18 | 5.7089E-17 |
| 539 | ATP5O | 0.614 | 0.784 | 2.38121E-07 | 3.70335E-07 |
| 10632 | ATP5L | 0.658 | 0.811 | 2.76927E-10 | 8.25324E-10 |
| 522 | ATP5J | 0.663 | 0.814 | 5.58777E-09 | 1.24556E-08 |
| 518 | ATP5G3 | 0.660 | 0.812 | 2.70222E-09 | 6.45981E-09 |
| 506 | ATP5B | 0.670 | 0.819 | 7.53437E-09 | 1.63897E-08 |
| 498 | ATP5A1 | 0.609 | 0.780 | 8.21153E-07 | 1.13057E-06 |
| 55856 | ACOT13 | 0.761 | 0.872 | 2.30504E-13 | 1.42251E-12 |
| 50 | ACO2 | 0.707 | 0.841 | 4.16025E-12 | 1.86547E-11 |
| 51100 | SH3GLB1 | 0.724 | -0.851 | 1.88065E-11 | 7.23931E-11 |
| 9467 | SH3BP5 | 0.636 | -0.798 | 4.63024E-10 | 1.31066E-09 |
| 65055 | REEP1 | 0.729 | -0.854 | 6.63955E-13 | 3.65934E-12 |
| 5894 | RAF1 | 0.628 | -0.792 | 8.27126E-09 | 1.78421E-08 |
| 5019 | OXCT1 | 0.621 | -0.788 | 1.35084E-10 | 4.33119E-10 |
| 373156 | GSTK1 | 0.610 | -0.781 | 2.9388E-06 | 3.58473E-06 |
| 2820 | GPD2 | 0.663 | -0.814 | 6.60308E-10 | 1.80674E-09 |
| 2731 | GLDC | 0.777 | -0.882 | 5.28942E-14 | 3.95954E-13 |
| 60492 | CCDC90B | 0.621 | -0.788 | 1.45319E-10 | 4.60751E-10 |
| 55326 | AGPAT5 | 0.692 | -0.832 | 2.65233E-11 | 9.85245E-11 |

CN

| **Gene ID** | **Gene Symbol** | **r^2^** | **r** | **p value** | **q value** |
| --- | --- | --- | --- | --- | --- |
| 7385 | UQCRC2 | 0.780 | 0.883 | 0.000186565 | 0.002436025 |
| 7374 | UNG | 0.736 | 0.858 | 0.001721987 | 0.00918292 |
| 7263 | TST | 0.790 | 0.889 | 0.000417084 | 0.003912645 |
| 7052 | TGM2 | 0.670 | 0.819 | 0.00490678 | 0.017234027 |
| 23424 | TDRD7 | 0.652 | 0.808 | 0.00151624 | 0.008512642 |
| 8801 | SUCLG2 | 0.628 | 0.793 | 0.003322154 | 0.013584269 |
| 6687 | SPG7 | 0.623 | 0.789 | 0.002273036 | 0.010885641 |
| 253512 | SLC25A30 | 0.712 | 0.844 | 0.000636055 | 0.005007732 |
| 79085 | SLC25A23 | 0.694 | 0.833 | 0.001972665 | 0.00999482 |
| 788 | SLC25A20 | 0.779 | 0.882 | 0.000627922 | 0.004972564 |
| 55037 | PTCD3 | 0.739 | 0.859 | 0.000927635 | 0.006234287 |
| 5663 | PSEN1 | 0.819 | 0.905 | 5.25755E-05 | 0.001145377 |
| 5625 | PRODH | 0.701 | 0.837 | 0.006649963 | 0.020924194 |
| 5519 | PPP2R1B | 0.849 | 0.921 | 2.08914E-05 | 0.000737472 |
| 23761 | PISD | 0.722 | 0.850 | 0.000533592 | 0.004497004 |
| 5298 | PI4KB | 0.610 | 0.781 | 0.004065163 | 0.015324069 |
| 10455 | PECI | 0.733 | 0.856 | 0.000730246 | 0.005433664 |
| 5166 | PDK4 | 0.875 | 0.935 | 3.44131E-05 | 0.000934811 |
| 5091 | PC | 0.885 | 0.941 | 6.62666E-06 | 0.000398569 |
| 115209 | OMA1 | 0.827 | 0.909 | 5.86514E-05 | 0.001210457 |
| 79731 | NARS2 | 0.614 | 0.784 | 0.012076165 | 0.030217665 |
| 4552 | MTRR | 0.681 | 0.825 | 0.00100785 | 0.006538662 |
| 9650 | MTFR1 | 0.627 | 0.792 | 0.002764568 | 0.012168021 |
| 63931 | MRPS14 | 0.603 | 0.776 | 0.002993487 | 0.012716474 |
| 54996 | MOSC2 | 0.630 | 0.794 | 0.00247982 | 0.011323556 |
| 4336 | MOBP | 0.685 | 0.828 | 0.002207236 | 0.010695544 |
| 4257 | MGST1 | 0.796 | 0.892 | 0.000161195 | 0.002209144 |
| 4170 | MCL1 | 0.602 | 0.776 | 0.00753037 | 0.022660666 |
| 4128 | MAOA | 0.682 | 0.826 | 0.009798533 | 0.026717707 |
| 51601 | LIPT1 | 0.760 | 0.872 | 0.000332183 | 0.003461897 |
| 9812 | KIAA0141 | 0.735 | 0.858 | 0.000362765 | 0.003657016 |
| 3712 | IVD | 0.687 | 0.829 | 0.001704863 | 0.00911493 |
| 3303 | HSPA1A | 0.633 | 0.795 | 0.002347253 | 0.011050345 |
| 3295 | HSD17B4 | 0.728 | 0.853 | 0.000475252 | 0.004183567 |
| 26275 | HIBCH | 0.617 | 0.786 | 0.00256001 | 0.011563813 |
| 50865 | HEBP1 | 0.622 | 0.789 | 0.007017023 | 0.021662752 |
| 3032 | HADHB | 0.829 | 0.910 | 5.29714E-05 | 0.001145377 |
| 3030 | HADHA | 0.708 | 0.842 | 0.001211847 | 0.00732712 |
| 3033 | HADH | 0.617 | 0.786 | 0.004875164 | 0.017180538 |
| 57678 | GPAM | 0.703 | 0.838 | 0.001580619 | 0.00874947 |
| 2760 | GM2A | 0.823 | 0.907 | 0.000324329 | 0.003403732 |
| 2752 | GLUL | 0.682 | 0.826 | 0.001086149 | 0.006875913 |
| 2747 | GLUD2 | 0.642 | 0.801 | 0.001858208 | 0.009721453 |
| 2746 | GLUD1 | 0.670 | 0.818 | 0.002386777 | 0.011101775 |
| 2653 | GCSH | 0.697 | 0.835 | 0.000981252 | 0.006433788 |
| 2631 | GBAS | 0.604 | 0.777 | 0.00337784 | 0.013731636 |
| 80303 | EFHD1 | 0.922 | 0.960 | 1.0386E-06 | 0.000194443 |
| 1808 | DPYSL2 | 0.755 | 0.869 | 0.000324143 | 0.003403732 |
| 1666 | DECR1 | 0.878 | 0.937 | 1.41577E-05 | 0.000605842 |
| 1727 | CYB5R3 | 0.694 | 0.833 | 0.007897344 | 0.023366314 |
| 1371 | CPOX | 0.638 | 0.799 | 0.001831423 | 0.009639039 |
| 1355 | COX15 | 0.609 | 0.780 | 0.006016537 | 0.019730517 |
| 25932 | CLIC4 | 0.746 | 0.864 | 0.000381168 | 0.003763721 |
| 1160 | CKMT2 | 0.859 | 0.927 | 0.000112313 | 0.001789521 |
| 55349 | CHDH | 0.620 | 0.788 | 0.014945404 | 0.034617447 |
| 63933 | CCDC90A | 0.694 | 0.833 | 0.000921556 | 0.006213353 |
| 51300 | C3orf1 | 0.666 | 0.816 | 0.001364364 | 0.0079468 |
| 665 | BNIP3L | 0.650 | 0.806 | 0.002056633 | 0.010272564 |
| 56898 | BDH2 | 0.798 | 0.893 | 0.00029369 | 0.003204052 |
| 596 | BCL2 | 0.726 | 0.852 | 0.00104312 | 0.006697037 |
| 275 | AMT | 0.723 | 0.850 | 0.000460023 | 0.004104861 |
| 501 | ALDH7A1 | 0.730 | 0.854 | 0.000606714 | 0.004876735 |
| 4329 | ALDH6A1 | 0.693 | 0.832 | 0.0011264 | 0.007004375 |
| 217 | ALDH2 | 0.742 | 0.861 | 0.000551404 | 0.004595408 |
| 205 | AK3L1 | 0.641 | 0.801 | 0.001986886 | 0.010032102 |
| 50808 | AK3 | 0.856 | 0.925 | 1.92598E-05 | 0.00070564 |
| 204 | AK2 | 0.606 | 0.778 | 0.003075597 | 0.012942216 |
| 85007 | AGXT2L2 | 0.813 | 0.901 | 8.8529E-05 | 0.001541701 |
| 51 | ACOX1 | 0.632 | 0.795 | 0.024448138 | 0.047126069 |
| 64746 | ACBD3 | 0.610 | 0.781 | 0.015038818 | 0.034767294 |
| 37 | ACADVL | 0.716 | 0.846 | 0.003359694 | 0.013693637 |
| 10449 | ACAA2 | 0.613 | 0.783 | 0.010406636 | 0.02760057 |
| 10975 | UQCR | 0.603 | -0.776 | 0.011044798 | 0.028640146 |
| 84790 | TUBA1C | 0.660 | -0.812 | 0.005009527 | 0.017454565 |
| 10376 | TUBA1B | 0.690 | -0.831 | 0.004456913 | 0.016244491 |
| 51499 | TRIAP1 | 0.647 | -0.804 | 0.004507399 | 0.016363015 |
| 84134 | TOMM40L | 0.724 | -0.851 | 0.000661117 | 0.005119109 |
| 56993 | TOMM22 | 0.666 | -0.816 | 0.015837249 | 0.035807759 |
| 441151 | TMEM151B | 0.822 | -0.907 | 5.28658E-05 | 0.001145377 |
| 29928 | TIMM22 | 0.770 | -0.877 | 0.004683724 | 0.016798617 |
| 10440 | TIMM17A | 0.668 | -0.817 | 0.005731705 | 0.019104615 |
| 26517 | TIMM13 | 0.903 | -0.951 | 2.95568E-06 | 0.000295123 |
| 26519 | TIMM10 | 0.614 | -0.784 | 0.023333859 | 0.0457217 |
| 293 | SLC25A6 | 0.633 | -0.796 | 0.002187283 | 0.010659071 |
| 115286 | SLC25A26 | 0.691 | -0.832 | 0.006318358 | 0.020292986 |
| 79751 | SLC25A22 | 0.733 | -0.856 | 0.000390661 | 0.003809619 |
| 9997 | SCO2 | 0.695 | -0.833 | 0.004367702 | 0.016052337 |
| 80142 | PTGES2 | 0.669 | -0.818 | 0.001230225 | 0.007403514 |
| 5518 | PPP2R1A | 0.699 | -0.836 | 0.000779025 | 0.005654485 |
| 23203 | PMPCA | 0.628 | -0.793 | 0.002105125 | 0.010410618 |
| 11331 | PHB2 | 0.618 | -0.786 | 0.002448324 | 0.0112479 |
| 5188 | PET112L | 0.711 | -0.843 | 0.000581799 | 0.004754239 |
| 4913 | NTHL1 | 0.655 | -0.809 | 0.002199365 | 0.010685617 |
| 8508 | NIPSNAP1 | 0.681 | -0.825 | 0.001013453 | 0.006566885 |
| 4724 | NDUFS4 | 0.627 | -0.792 | 0.012725113 | 0.03122949 |
| 4720 | NDUFS2 | 0.638 | -0.799 | 0.017753161 | 0.038487871 |
| 4704 | NDUFA9 | 0.673 | -0.820 | 0.004032658 | 0.01525771 |
| 51537 | MTP18 | 0.629 | -0.793 | 0.002710105 | 0.012022764 |
| 51081 | MRPS7 | 0.700 | -0.837 | 0.00660708 | 0.020839755 |
| 6183 | MRPS12 | 0.627 | -0.792 | 0.010577483 | 0.027842391 |
| 51073 | MRPL4 | 0.627 | -0.792 | 0.003293607 | 0.013493854 |
| 51253 | MRPL37 | 0.726 | -0.852 | 0.001687105 | 0.009058593 |
| 6150 | MRPL23 | 0.629 | -0.793 | 0.002373185 | 0.011078997 |
| 64928 | MRPL14 | 0.641 | -0.801 | 0.003016592 | 0.012778383 |
| 6182 | MRPL12 | 0.760 | -0.872 | 0.000313127 | 0.003332341 |
| 28992 | MACROD1 | 0.841 | -0.917 | 0.00568904 | 0.019007306 |
| 84908 | FAM136A | 0.746 | -0.864 | 0.001199901 | 0.007296969 |
| 51295 | ECSIT | 0.781 | -0.884 | 0.000261262 | 0.003026646 |
| 55794 | DDX28 | 0.617 | -0.785 | 0.003632525 | 0.014262919 |
| 1537 | CYC1 | 0.679 | -0.824 | 0.002864573 | 0.012430753 |
| 1508 | CTSB | 0.718 | -0.847 | 0.005250204 | 0.01795892 |
| 126129 | CPT1C | 0.690 | -0.831 | 0.000991721 | 0.006478121 |
| 9377 | COX5A | 0.657 | -0.811 | 0.023959846 | 0.046570144 |
| 1352 | COX10 | 0.726 | -0.852 | 0.000455554 | 0.004104861 |
| 91647 | ATPAF2 | 0.603 | -0.776 | 0.005037367 | 0.017516639 |
| 2182 | ACSL4 | 0.662 | -0.814 | 0.00654101 | 0.020699404 |
| 11332 | ACOT7 | 0.654 | -0.809 | 0.006695033 | 0.021013638 |
