# Supplementary material for: Expression Profiles of Mitochondrial Genes in the Frontal Cortex and the Caudate Nucleus of Developing Humans and Mice Selectively Bred for High and Low Fear
Source: PLoS One. 2012 Nov 13;7(11):e49183. doi: 10.1371/journal.pone.0049183 (PMC3496717; doi:10.1371/journal.pone.0049183)
Supplement: Table S2 — Information on the genes associated with the GO term: mitochondrion in the PFC and the CN. r2: adjusted coefficient, r: regression coefficient, q value: FDR-adjusted q-value (DOCX) [file pone.0049183.s004.docx]

Table S2. A summary of demographic information.

PMI: Postmortem interval, RIN: RNA integrity number

M: Male, F: Female, AA: African American, C: Caucasian

| **Sample** | **Age (year)** | **Age Group** | **Sex** | **Race** | **PMI** | **Brain pH** | **RIN** | **Brain Region** |
| --- | --- | --- | --- | --- | --- | --- | --- | --- |
| 1 | 0.11 | Neonate | M | AA | 27 | 6.47 | 8.9 | PFC |
| 2 | 0.15 | Neonate | M | AA | 17 | 6.63 | 9.5 | PFC |
| 3 | 0.15 | Neonate | M | C | 11 | 6.86 | 9.1 | PFC |
| 4 | 0.17 | Neonate | F | AA | 27 | 6.52 | 8.6 | PFC |
| 5 | 0.19 | Neonate | M | AA | 25 | 6.5 | 8.6 | PFC |
| 6 | 0.21 | Neonate | M | AA | 28 | 6.6 | 9.1 | PFC |
| 7 | 0.24 | Neonate | F | AA | 24 | 6.65 | 8.9 | PFC |
| 8 | 0.25 | Infant | F | AA | 14 | 6.54 | 9.1 | PFC |
| 9 | 0.32 | Infant | M | C | 19 | 6.36 | 9.1 | PFC |
| 10 | 0.33 | Infant | M | C | 22 | 6.54 | 8 | PFC |
| 11 | 0.35 | Infant | M | C | 27 | 6.66 | 8.2 | PFC, CN |
| 12 | 0.38 | Infant | M | AA | 9 | 6.54 | 7.5 | PFC |
| 13 | 0.39 | Infant | M | AA | 5 | 6.81 | 9.4 | PFC, CN |
| 14 | 0.52 | Infant | F | AA | 22 | 6.82 | 9 | PFC |
| 15 | 0.54 | Infant | M | AA | 24 | 6.71 | 9 | PFC |
| 16 | 0.91 | Infant | M | AA | 18 | 6.87 | 8.6 | PFC, CN |
| 17 | 1.58 | Toddler | F | C | 24 | 6.9 | 8.2 | PFC |
| 18 | 2.19 | Toddler | M | AA | 27 | 6.64 | 8.2 | PFC |
| 19 | 2.45 | Toddler | F | AA | 22 | 6.74 | 8.3 | PFC, CN |
| 20 | 2.71 | Toddler | F | C | 44 | 6.47 | 7.4 | PFC |
| 21 | 4.64 | Toddler | M | C | 18 | 6.92 | 7.9 | PFC |
| 22 | 4.86 | Toddler | M | AA | 19 | 6.74 | 9.1 | PFC |
| 23 | 5.39 | School age | M | C | 17 | 6.74 | 8.6 | PFC |
| 24 | 8.00 | School age | F | C | 20 | 6.78 | 9.3 | PFC |
| 25 | 8.00 | School age | M | AA | 5 | 6.76 | 8.3 | PFC |
| 26 | 11.54 | School age | F | C | 12 | 6.44 | 8.6 | PFC |
| 27 | 12.42 | School age | M | C | 16 | 6.82 | 8.2 | PFC, CN |
| 28 | 12.97 | School age | F | C | 18 | 6.85 | 8.9 | PFC, CN |
| 29 | 15.00 | teenage | M | AA | 13 | 6.76 | 6.2 | PFC |
| 30 | 16.68 | teenage | F | C | 16 | 6.81 | 7.8 | PFC |
| 31 | 17.05 | teenage | M | C | 25 | 6.69 | 8.6 | PFC, CN |
| 32 | 17.38 | teenage | M | C | 19 | 6.84 | 7.5 | PFC, CN |
| 33 | 17.69 | teenage | M | AA | 16 | 6.83 | 8.5 | PFC |
| 34 | 17.82 | teenage | M | C | 12 | 6.8 | 9.2 | PFC |
| 35 | 20.14 | Young adult | M | AA | 18 | 6.5 | 8.6 | PFC |
| 36 | 21.93 | Young adult | M | C | 13 | 6.96 | 8.6 | PFC |
| 37 | 22.92 | Young adult | M | AA | 4 | 6.84 | 9.4 | PFC, CN |
| 38 | 23.62 | Young adult | F | AA | 14 | 6.57 | 9.1 | PFC |
| 39 | 24.93 | Young adult | M | C | 7 | 6.92 | 9.3 | PFC, CN |
| 40 | 25.38 | Young adult | F | C | 16 | 6.73 | 9.2 | PFC, CN |
| 41 | 35.99 | Adult | M | C | 13 | 6.73 | 8.7 | PFC |
| 42 | 38.42 | Adult | F | AA | 19 | 6.98 | 8.2 | PFC |
| 43 | 38.63 | Adult | M | AA | 8 | 6.37 | 8.2 | PFC, CN |
| 44 | 42.94 | Adult | M | C | 18 | 6.49 | 8.2 | PFC |
| 45 | 46.18 | Adult | M | AA | 18 | 6.75 | 8.1 | PFC, CN |
| 46 | 49.22 | Adult | F | AA | 7 | 6.78 | 7.4 | PFC |
